# A novel *ε*-sensitive correlation indistinguishable scheme for publishing location data

**DOI:** 10.1371/journal.pone.0226796

**Published:** 2019-12-19

**Authors:** Wang Bin, Zhang Lei, Zhang Guoyin

**Affiliations:** 1 College of Computer Science and Technology, Harbin Engineering University, Harbin, PR China; 2 College of Information Science and Electronic Technology, Jiamusi University, Jiamusi, PR China; Victoria University, AUSTRALIA

## Abstract

Nowadays, location based service (LBS) is one of the most popular mobile apps and following with humongous of location data been produced. The publishing of location data can provide benefit for promoting the quality of service, optimizing the commercial environment as well as harmonizing the infrastructure construction. However, as location data may contain some sensitive or confidential information, the publishing may reveal privacy and bring hazards. So the published data had to be disposed to protect the privacy. In order to cope with this problem, a number of algorithms based on the strategy of *k*-anonymity were proposed, but this is not enough for the privacy protection, as the correlation between the sensitive region and the background knowledge can be used to infer the real location. Thus, consider about this condition, in this paper a *ε*-sensitive correlation privacy protection scheme is proposed, and provides correlation indistinguishable to the location data. In this scheme, entropy is first used to determine the location centroid of each cell to build up the voronoi diagram. Then the coordinate of the untreated location data that is located in the cell is transferred into the centroid vicinity. Accordingly, the sensitive correlation is destroyed by the coordinate of each published data. The process of transferring the location data is determined by metrics of *ε*-sensitive correlation privacy, and is rigorous in mathematical justification. At last, security analysis is proposed in this paper to verify the privacy ability of our proposed algorithm based on voronoi diagram and entropy, and then we utilize the comparative experiment to further affirm the advantage of this algorithm in the location data privacy protection as well as the availability of published data.

## Introduction

For the past few years, with the development of mobile communication technology, location based service (LBS) has become more important in people's daily life, and more and more people became to enjoy the convenience of this type of service. Along with the service, humongous of location data is produced and collected by the service provider. Then the data is published to organizations such as scientific research institution, government departments as well as the commercial enterprise, so as to develop the correlation technique, the policies and regulations and the commercial popularizing. However, location information is important to the user, as it may contain sensitive or confidential information [1–3]. So the publishing of location data may reveal the location privacy of the user, especially for the publishing of locations in the vicinity of the hospital or consuming place.

In order to cope with the problem of privacy disclosure in the publishing of location data, several works had been proposed in the past few years. In 2010, Fung et al.[4] had given a survey on the development situation of privacy protection in data publishing, and in the survey they classified the scheme of publishing data protection as suppression and generalization. They also thought, as a particular type of publishing data, location data contains the sensitivity of itself as well as the sensitivity to correlate other sensitive information. For one thing, location information may reveal the behavior privacy of the user in daily habits, such as the routing of morning exercises, the favorite supermarket and so on. For another, it can be used to infer other sensitive locations, such as the home address, the workplace, even the entertainment venues. So the treatment of location data will be different from other data in privacy protection. To cope with the privacy of location data publishing, Xu et al.[5] considered the transformation of sensitive locations. They thought that if the sensitive location is removed from the set of publishing data, the privacy of the user will be protected. But they failed in cutting off the correlation between the private location and the background knowledge of sensitive location. The correlation can be used by the adversary to correlate with the published information to infer the privacy of the user. Bonchiet al.[6] regarded the location privacy as the process of hiding the routing in location trajectory. They obfuscated the intermediate nodes of the trajectory and published the data that just contains the information of origination and destination, so the data of the whole routing is obfuscated. But most of the time, origination and destination locations are just sensitive locations of the user. Sui et al.[7] took care of the parking location in the user's movement, and utilized the spatio-tenporal tunnel to transmit the parking location into a similar sub-trajectory to obfuscate the publishing data. Zhao et al.[8]suppressed the information of the sensitive location in the routing of the user. They also considered the correlation between the publishing location and the sensitive location, and suppressed the correlation data with the occurrence frequency. Wang et al.[9] provided a Markov prediction based scheme in traffic monitor of data of publishing, and utilized the stretch of predicted road to reduce the correlation between the publishing location and the sensitive location.

No doubt that, these algorithms had provided privacy protection service to location data in publishing. However, with increasing background knowledge grasped by the adversary, he can utilize the relation between the published location and sensitive location to correlate the location privacy, which made the primary strategy based on *k*-anonymity became no longer efficiency in measuring privacy. Moreover, these algorithms often made vast transformation on the prepared data, and reduced the availability of published data. Thus, a scheme can provide private service for the publishing of location data, resist the attack of sensitive location correlation, slip the leash of *k*-anonymity and provide higher data availability as needed. In this paper, we had attempted to solve above problems and proposed a *ε*-sensitive correlation indistinguishable scheme with differential privacy. In this scheme, the feature of location data is considered firstly, and we had found that the location which has the similar probability to correlate all sensitive locations is difficult to be correlated by the adversary. Furthermore, locations with similar attributes could be substituted by a location in the same set without or rarely affecting the availability. Then based on above foundation, we provide an algorithm with entropy and voronoi diagram (short for E-V) to achieve the *ε*-sensitive correlation indistinguishable. At last, we had proposed security analysis in this paper, and had a detailed description on how the E-V provides privacy protection service for publishing location data. Results of comparative experiments with detailed interpretations are shown to further affirm the advantage of this algorithm in location data privacy protection as well as the availability of published data. Thus, based on security analysis and comparative experiment, we can consider that our proposed E-V algorithm with *ε*-sensitive correlation indistinguishable is suitable for application in the realistic environment and has vast potential for future development.

The contribution of this paper is mentioned as follows.

- We proposed a *ε*-sensitive correlation indistinguishable scheme with differential privacy, and utilized the *ε*-sensitive correlation indistinguishable to measure the degree of coordinate adjacency, so the transmitted location can protect the privacy and provide minor influence on data availability.
- We provided an algorithm with entropy and voronoi diagram (short for E-V) to achieve the ε-sensitive correlation indistinguishable.
- We experimentally evaluate the proposed scheme with the actual data, the analytical and simulation results show that our scheme can achieve the objectives efficiently.

The rest of this paper is organized as follows. Firstly, we introduced the related work in Section 2. Section 3 presents the process of publishing data together with security analysis. Then we show experimental results and cause analysis in Sections 4. Finally, we draw conclusions in Section 5.

## Related works

Based on the use-pattern of location data, the proposed privacy protection algorithms can be classified into two types. The first one is designed for location based service in location protection of real time. The other is designed for the location data which is used for publishing.

To algorithms of real time location data protection, *k*-anonymity [10] is the most used strategy. They utilized a trusted third party called central server to find at least *k*- 1 other users to generalize the real location of the user, and send the real location with the generalized location to the LBS server, so the adversary cannot identify the specified user in an anonymous location set. Since then, other schemes such as the semantic *p*-sensitivity [11] and the query *l*-diversity [12] had evolved from the conception of *k*-anonymity. In recent years, researchers have began to pay close attention to the generalization of location trajectory in continuous query[13, 14], the privacy protection scheme without central server [15–17], as well as the correlation in attributes during the routing of continuous query[18–21],others include the schemes of encryption[22–24].

Privacy protection algorithms in the publishing of location data were mainly concentrated on suppression and generalization. In general, suppression is used to get rid of the data that concerned with the potential privacy of the user. The process of suppression is based on the sensitive attribute, the isolated position or the relational degree of publishing data. In this type, the most important scheme is proposed by Chen et al.[25]. In their scheme, a (*K*,*C*)_*L*_ privacy protecting model is proposed based on *k*-anonymity. They thought that, although the adversary had grasped at least *L* locations in the moving trajectory, if and only if the suppressed data had the correlation less than *C*, the adversary still cannot get any privacy of the user through the location correlation. Then Terrovitis et al.[26] expanded this scheme with local suppression. In their scheme, the suppressed target is no longer the global data but just the data item, so the suppression amount of the data is reduced and improved the availability of published data. However, although these schemes had provided privacy protection for location data, the suppression no matter global or local had destroyed the relativity of the published data, it makes the customer hardly to finding useful knowledge, let alone the better quality of servicing, commercial environment optimizing or infrastructure construction harmonizing. Thus, researchers had to divert their attention to find another scheme with better availability and equivalent privacy protection level, and then they anchored their hope on generalization.

The strategy of generalization is different from suppression. In generalization, the published data are divided into groups with similar data or added dummy data to achieve k-anonymity, so the adversary cannot identify a particular user in anonymous set. As the transformation of the original data is minimum, the data proceed by generalization had a better availability and a parallel privacy protection level, so it makes the algorithms of this type used widely and practically. In the early days, clustering is used in finding the data with similar attributes. Bonchi et al. [6] utilized the similar features of clustering location data to generalize the privacy of a particular user, and verified that the generalized data is difficult to be attached to any specified user. Like the strategy with Bonchi, Chow et al. [27] provided a hierarchy extending scheme. In their scheme, the region of publishing location is extended to contain more locations. Then the locations set will contain similar kind of data that can generalize with each other, so the privacy of a particular user will be protected. However, in currently, with the development of data mining and data analysis, more and more methods and attributes can be used in discovering the potential correlation between any published data. Simultaneously, the requirement of individualization also affects the daily life of the user. Thus, the privacy protection scheme has to confront both of the infer attack as well as individualization service. Lu et al. [28] considered the individualization requirement of the user and added personal privacy criterion in the process of data publishing. Zheng et al. [29, 30] found that the attributes information in published data can be used to correlate the user, and they provided their scheme with attributes generalization. Li et al. [31] assumed the adversary can obtain the maximum degree of background knowledge to initial the differential attack, and they had utilized the principle of differential privacy to provide a dummy scheme to protect the privacy. There is no doubt that, the series of current algorithms had improved the capacity of location data privacy protection in data publishing.

However, just as we have analyzed in the first section, there is still no scheme that can provide private service, resist the attack of correlation, slip the leash of k-anonymity and provide higher data availability simultaneously. Thus, in this paper, we had proposed a *ε*-sensitive correlation indistinguishable scheme with differential privacy, entropy as well as voronoi diagram. We also expected that this scheme can be used as the supplement of current algorithms, and been applied in practical application.

## The process of publishing data

The *ε*-sensitive correlation indistinguishable is implemented by the transmission of location data, and this transmission is based on the feature of voronoi diagrams. Thus, two steps are needed to process location data in our proposed scheme. In the first step, entropy is used to measure the correlation between the location and vicinity sensitive locations. Then the location that has the highest entropy is chosen as a candidate and stored in a stated set. In the second step, a voronoi diagram is established with locations in the stated set as the centroid of each cell. At last, location data prepared for publishing in the cell are transmitted into the coordinate of centroid, and then published to the data customer. In this algorithm, the correlation between the published location and the sensitive location is destroyed by entropy, as the published location has the similar probability to be correlated to any sensitive position. Furthermore, the feature of voronoi diagram is utilized. As the distance between the centroid and the location in the same cell is the shortest one than the distance to the other centroid. So this feature guaranteed the highest availability of the published data. Under the procedure of E-V, we can see that the parameter *ε* is determined by entropy, and the higher of entropy the lower of *ε*.

## The centroid chosen with entropy

The strategy of E-V maybe similar to the clustering scheme, as both of them all has to find the centroid or center of the clustering. In our scheme, the E-V algorithm has to utilize the centroid to replace the location in the same cell, so randomly chosen the centroid is not appropriate. Because of that the arbitrary centroid may be the sensitive location or can be easy to be correlated to a sensitive location (such as hospital, bank or bars). Thus, entropy is calculated by the probability of the centroid correlated to the sensitive location and used in the selection of centroid. Assume *l*_v_ is the location of centroid and the sensitive location set is denoted as *L*_s_ = (*l*_s1_,*l*_s2_,…,*l*_s*n*_), where is *n* the number of sensitive locations in the vicinity of *l*_v_. Then the probability between the centroid and sensitive location can be denoted as $p_{i}=\frac{l_{v}\to l_{i}}{l_{v}\to L_{s}},0\leq i\leq n$. With the probability *p*_*i*_, entropy to express the degree of current centroid location can be denoted as

$$H=-\sum_{i=1}^{n}p_{i}\log_{2}p_{i}$$ (1)

Based on the Jaynes’ rationale on maximum entropy methods, we can affirm that, if and only if *p*_1_ = *p*_2_ = … = *p*_*n*_, the maximum entropy $H_{\max}=-\frac{1}{n}\log_{2}\frac{1}{n}$ can be calculated. It means for an arbitrary *l*_*i*_ and *l'*_*i*_ in the sensitive location set *L*_s_, the correlation probability between the centroid location *l*_v_ and *l*_*i*_ is equal to the probability between *l*_v_ and *l'*_*i*_. Accordingly, the probability values of *l*_v_ to *L*_s_ are equal to each other. Then the adversary cannot identify any particular user in the published data through the correlation attack, and the privacy of the user is protected.

As the voronoi diagram has multiple cells, the centroid for each cell has to be calculated. Assume the set of centroid is denoted as *L*_v_ = (*l*_v1_,*l*_v2_,…,*l*_v*m*_), where *m* is the number of centroid in the region of the publishing data, so the correlation probability for each centroid in the voronoi diagram has to be calculated. In Algorithm 1, the procedure of centroid chosen with entropy is given.

**Algorithm 1** The centroid chosen with entropy

**Input:**The set of centroid locations *L*_v_, the set of sensitive locations *L*_s_

**Output:**The chosen centroid locations *L*_vu_

1) for(*i* = 1; *i*< = *m*; ++*i*)

2)   for(*j* = 1; *j*< = *n*; ++*j*)

3) $p_{j}=\frac{l_{i}\to l_{j}}{l_{i}\to L_{s}}$;//calculate the correlation probability between the centroid and the sensitive locations.

4) $H_{i}=-\sum_{j=1}^{n}p_{j}\log_{2}p_{j}$;// calculate the entropy with the current centroid.

5)     if(*H*_*i*_ = = max)

6) *L*_vu_ = *l*_*i*_;// add the current location into centroid set.

7)       else

8)         break;

9)       end if

10)   end

11) end

Under above two loop operations, the centroid set is established with the help of entropy, so the time complexity will be *O*(*n*^2^). In the line of 3–4 in Algorithm 1, the correlation probability between the centroid and the sensitive locations is calculated, and then the value of entropy is calculated with these probability values. Then in the line of 5–9, the value is estimated by the maximum value of entropy, and the location that satisfies the condition is chosen as the candidate and stored in the centroid set. After the procedure of centroid is chosen, the voronoi diagram can be established based on the chosen set, and then the sequential step is to transmit the original data into the centroid.

## The transmission of publishing data with centroid

**Fig 1:**
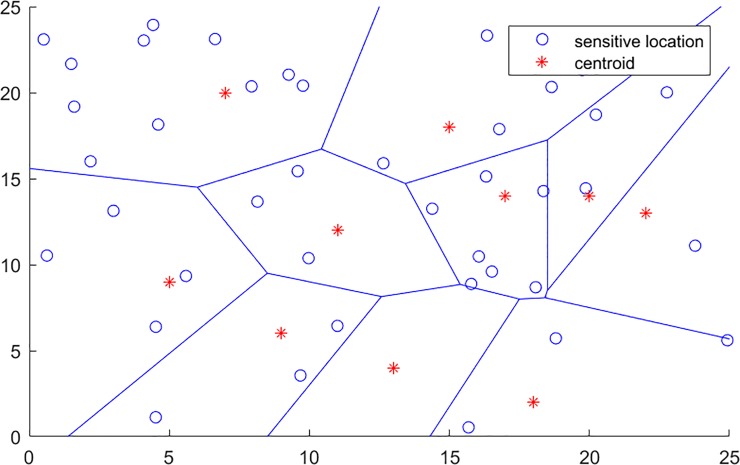
The region divided by voronoi diagram.

Before the procedure of coordinate transmission for location data, the region and voronoi diagram of publishing data has to be established firstly. Consider that, there is a region contains publishing data about somewhere, and the randomly chosen cell number is eight, then this region can be denoted as Fig 1. In this figure, the sensitive locations are labeled as circular rings, the calculated centroid in each cell is labeled as *, the region is divided by the solid line and formatting multiple polygons. As the coordinate of each node can be obtained from this region, above process can be achieved by Algorithm 1. After the procedure of centroid is chosen and voronoi diagram establishing, the publishing coordinates in each cell of the voronoi diagram had to be transmitted into the coordinate of the centroid.

In general, it is the best to transmit the original data into the centroid coordinate, as it can provide the best privacy protection. However, under such circumstances, the availability of published data will be dropped to the minimum value. Then the balance between privacy protection and data availability will be broken, and there is no or less useful to the published data. Thus, we need to provide a measure criterion to maintain the balance between privacy and availability, so we utilize the conception of Zhang et al. [19]used for the indistinguishable of correlation probability. Consider that, if the specified centroid coordinate is denoted as *l*_v_ and the set of sensitive locations is denoted as *L*_s_ = (*l*_s1_,*l*_s2_,…,*l*_s*n*_), then the correlation probability satisfied the probability function *K*(*L*_*s*_,*l*_*v*_)→*P*(*Z*), where *Z* is the set of reported value. Thus, the similarity of probability distribution can be defined by the metric *M*_*P*_(*μ*_1_,*μ*_2_) = *Sup*_*z*∈*Z*_|*μ*_1_(*z*)-*μ*_2_(*z*)|. If both *μ*_1_(*z*) and *μ*_2_(*z*) equals to zero or ∞, |*μ*_1_(*z*)-*μ*_2_(*z*)|. In other words, *M*_*P*_(*μ*_1_,*μ*_2_) is small if *μ*_1_,*μ*_2_ assign similar probabilities to each value *z*.

The metric *M*_*P*_ denotes the indistinguishable level between *p* and *p*', the smaller of this value the more degree of indistinguishable between the centroid coordinate and the sensitive location. If it exceeds a certain value, we can confirm that the adversary can distinguish the probabilities produced by uncertain locations, and correlate any specified location with the sensitive location. Thus, we have the definition of *ε*-sensitive correlation differential privacy under the metric *M*(*p*,*p*').

The definition of *ε*-sensitive correlation indistinguishable can be denoted as that, if *l*_*s*_ and *l*'_s_ are two random locations selected from the set of sensitive location *L*_s_ = (*l*_s1_,*l*_s2_,…,*l*_s*n*_), a mechanism *P*(*K*(*L*_*s*_),*l*_*v*_)→*P*(*Z*) can satisfy the *ε*-sensitive correlation indistinguishable if and only if

$$M_{P}(p(K(l_{s}),l_{v}),p(K(l_{s}^{'}),l_{v}))\leq \varepsilon M_{P}(p(l_{s},l_{v}),p(l_{s}^{'},l_{v}))$$ (2)

Equivalently, this definition can be formulated as $K(l_{s},l_{v})(z)\leq e^{\varepsilon M_{P}(p(l_{s},l_{v}),p(l_{s}^{'},l_{v}))}K(l_{s}^{'},l_{v})(z)$ for all *z*∈*Z*. The privacy parameter *ε* can be seen as the scaling of the metric *M*_*P*_.

With the constraint of parameter *ε*, data availability can be selected under the acceptable privacy deficiency. Thus, we can utilize coordinates in the vicinity of the centroid of voronoi cell to instead of just the coordinate of centroid, and give back the availability to the published data. The parameter *ε* can be calculated by *ε* = |*l*_*v*_−*l*_*vi*_|, where *l*_*vi*_ is the location in the vicinity of the centroid. Then the procedure of transmitting the original data into the published data can be denoted as algorithm 2, and this process is denoted as follows. Where *L*_vu_.num denotes the number of the data in the set centroid coordinate in voronoi diagram, and *l*_*iv*_.co denotes the location that may be correlated by the adversary.

**Algorithm 2** The procedure of transmitting the publishing data

**Input:**The set of publishing data *L*, the set centroid coordinate in voronoi diagram *L*_vu_

**Output:**The set of published data *L'*

1) Divide the original data *L* with the cell of voronoi diagram under the constraint of centroid set *L*_vu_;

2) for(*j* = 1; *j*< = *L*.num; ++*j*)//the coordinate of publishing data.

3) for(*i* = 1; *i*< = *L*_vu_.num; ++*i*)

4)     R(*i*)∈the region of cell(*i*); //establishing the cell of voronoi diagram.

5) *l*_*iv*_.co = | *l*_*i*_.co-*ε*|;// calculate the transmitted location with parameter *ε*.

6)     if(*l*_*j*_∈R(*i*))

7) *l*_*j*_.co = *l*_*iv*_.co; //transmit the publishing location to coordinates in the vicinity of the centroid.

8)     else

9)         continue;

10)       end if

11) end

12) end

In Algorithm 2, original data is transmitted into coordinates of the vicinity of centroid in each voronoi cell, and the published data are restrained under the definition of *ε*-sensitive correlation indistinguishable. The line of 3–10 is the procedure of transmitting each data into the published data, and the line 6–9 is the judgment of whether the current location belongs to the certain cell, so the transmission can be executed successfully. As there is also a two loop operations, the time complexity for algorithm 2 will similar with algorithm and equals to be *O*(*n*^2^)

## Security analysis

For the security of published data, there are two phases to guarantee the privacy of the user, the first one is the entropy used as the privacy metric that guarantees the adversary has equal probability to correlate the centroid; the other is correlation indistinguishable which guarantees the adversary cannot infer the real location even if her gains the background knowledge.

Firstly, all the locations in sensitive in the published data are changed into the centroid, which means any arbitrary location *l*_*i*_ is changed into the centroid *l*_v_, so the probability to correlate any *l*_*i*_ with *l*_v_ will be equal with each other and *p*_1_ = *p*_2_ = … = *p*_*n*_, then the maximum value of entropy $H_{\max}=-\frac{1}{n}\log_{2}\frac{1}{n}$ can be calculated, which means the adversary will have the most uncertainty about the background knowledge he gained and will be difficult to correlate any locations in sensitive.

Secondly, with the help of transmitting the original data into coordinates of the vicinity of centroid in each voronoi cell, the published data will preserve the privacy of the user. This operation is determined by the indistinguishable of probability between the centroid and sensitive location. According to the definition of *ε*-sensitive correlation indistinguishable, we can consider that, the smaller of parameter *ε* the higher level of published data. Thus, the private level of the published data is determined by *ε*. Assume that, there is a publishing data located in the vicinity sensitive location *l*_s_, and this location also located in the cell of established voronoi diagram. As the centroid of each cell in the voronoi diagram satisfied the constraint of each correlation probability equals to each other and the maximum entropy. Thus, we can consider that, for another randomly chosen sensitive location *l'*_s_, the correlation probability of *p*(*l*_*s*_,*l*_*v*_) equals to $p(l_{s}^{'},l_{v})$. Then with the published data *l*_*v*_ the adversary cannot obtain any information about the user under the attack of sensitive probability correlation, because he cannot distinguish the difference between two probability values, as the difference is less than *ε*. With the two randomly chosen sensitive location *l*_s_ and *l'*_s_, Eq (2) can be changed into

$$\begin{array}{r} K(l_{s},l_{v})(z)\leq e^{\varepsilon M_{P}(p(l_{s},l_{v}),p(l_{s}^{'},l_{v}))}K(l_{s}^{'},l_{v})(z)\\ =e^{\varepsilon M_{P}(p(l_{s},l_{v}),p(l_{s},l_{v}))}K(l_{s}^{'},l_{v})(z) \end{array}$$ (3)

As *M*_*P*_(*p*(*l*_*s*_,*l*_*v*_),*p*(*l*_*s*_,*l*_*v*_))≠0, if $K(l_{s},l_{v})(z)=K(l_{s}^{'},l_{v})(z)$ then *ε* must be equals to zero. In this paper, the published location is transmitted into the coordinate of the vicinity of *l*_*v*_, and the correlation probability differential is smaller than *ε*. Thus, we can consider that $K(l_{s},l_{v})(z)=K(l_{s}^{'},l_{v})(z)$ can be converted to *K*(*l*_*v*_,*l*_*v*_)(*z*) = *K*(*l*_*v*_,*l*_*v*_)(*z*). It means that, the data processed by algorithm E-V have the sensitive correlation indistinguishable under the differential of smaller than *ε*, and this differential cannot be distinguished by the adversary. At last, the privacy of the user is protected and the published data are secure.

## Experimental evaluation

### Experiments setup and evaluation criteria

The data set used in the experimental evaluation is collected from the traveling trajectory of a Beijing taxi. The data of Beijing taxi is collected by the smart phone with GPS ID of 4168722 in 15 days by an author along with the driver who does not belong to any company and the plate number of this taxi we had to conceal and we had paid him in these days for buying the data. This data is mainly collected by the author, so we have the permission to share this data. In this set, the sensitive locations are chosen from the landmark of the scenic area, bank or other administrative bodies. Then the experiment is simulated by Matlab R2017a and implemented on a laptop with Intel Core i5 2.40 GHz CPU, 4 GB RAM memory and Windows 7×64 ultimate operating system. In order to further verify the privacy protection ability and guarantee the availability of published data, a series of simulation experiments are proposed. In the simulation experiment, several similar algorithms had been compared (such as the privacy protection scheme with local suppression KCL-Local[25], the obfuscated group p-confidentiality[32], as well as the clustering based ECC[33]). Then based on the general character of these schemes, we formulated the following evaluation criteria in both of the privacy protection ability and the availability of published data.

As the difference of background knowledge of the adversary grasped, the relevance between the published location and the sensitive location is defined as the connectedness of both points. Thus, the probability of correlation is calculated by the connectedness of two locations in the vicinity, and entropy is used as metrics to measure the privacy of the published data. Furthermore, the ratio of privacy processing is another criterion used to verify the privacy protection ability, as the more information been processed under the algorithm means the higher privacy level of the published data. Then, in order to measure the availability of published data, three different criteria are proposed. They are the number of association rules mining from the published data, the shift distance of published location from the original location as well as the ratio of data retention.

In the comparison of privacy protection, we consider the correlation between the publishing location and the sensitive location is *p*(*i*), then the formulation of entropy can be denoted as

$$H(i)=-\sum_{i=1}^{n}p(i)\log_{2}p(i)$$ (4)

If the probability value equals to the others, it means the maximum entropy can be acquired and the adversary cannot utilize the correlation attack to guess the privacy of the user. Therefore, the larger of entropy value means the higher of privacy level.

The ratio of privacy processed can be considered as the ratio of private information was processed. In this paper, we consider the location which can be attached to the sensitive location as the private location. Assume that the number of unprocessed location is I_f_ and the number of processed location is I_a_. Thus, the ratio of privacy processed can be denoted as

$$P(I)=\frac{I_{f}^{}{‐I}_{a}^{}}{I_{f}^{}}$$ (5)

In the aspect of published data available, the number of association rules is calculated by the Apriori algorithm [34], and this number is ascending with the number of published data.

The shift distance of published location is calculated by the difference of the published location minus the original location, so the longer of this distance means the less availability of published data. Consider the set of published location is L_a_ and the set of original location is L_a_, then the shift distance can be denoted as

$$D_{m}{=|L}_{a}{‐L}_{f}|=\sum_{i=1}^{n}{|l}_{ai}{‐l}_{fi}|$$ (6)

At last, the ratio of data retention is calculated by the ratio of the amount of published data and the original data. If the ratio is greater than a certain value, it means the published data is not applicable. Assume the amount of published data is D_a_ and the amount of original data is D_f_, then the ratio of data retention can be denoted as

$$D_{l}=\frac{D_{f}-D_{a}}{D_{f}}$$ (7)

In the following section, the evaluation results with the reason analysis will be given.

## Evaluation results and cause analysis

**Fig 2:**
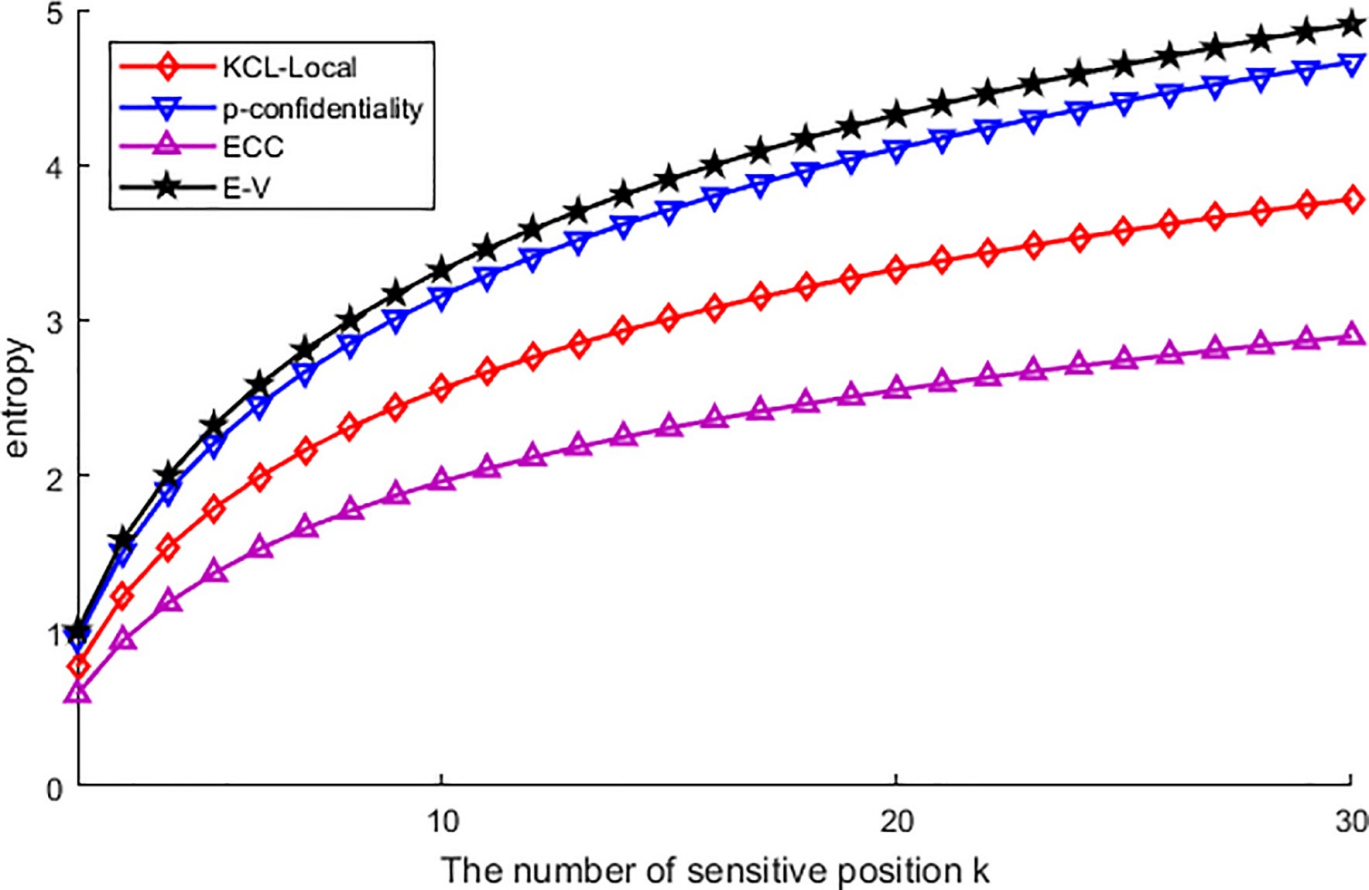
Entropy of the uncertainty.

Fig 2 shows the uncertainty of the adversary guesses the privacy of a particular user, and this uncertainty is measured by entropy. From this figure, the uncertainty of published data is ascending with the increasing number of sensitive locations. This is because of that, with the increase of sensitive location, the adversary becomes difficult to correlate the published location to any sensitive position, and the increasing number of sensitive locations improved the uncertainty. In turn, the value of entropy became increasing gradually. Among the performance of all algorithms, we have found the E-V is the best one to achieve the maximum entropy along with the increase of sensitive position. As the privacy of this algorithm is measured by ε-sensitive correlation indistinguishable, this made the published data have the similar correlation probability to each other. The similarity feeds back the indistinguishable to any of the published data, though the correlation attack is used by the adversary. For the other three algorithms, p-confidentiality performs better than the other two. This is because this algorithm has transmitted sensitive locations into an obfuscated region. In this region the correlation between the published location and sensitive position has been obfuscated. Then obfuscation makes the adversary difficult to correlate the special user through correlation attack. But as this scheme just chosen the region without considering the similarity of correlation probability, there still retained some correlation can be used by the adversary, so it is slightly inferior. The KCL-Local utilizes the suppression to provide privacy protection, which means that this algorithm does not consider the correlation between the published location and the sensitive position, and the correlation is cut off just as some correlation data are got rid of by suppression. Thus, the adversary still can utilize the correlation attack to get the privacy of a particular user. At last, the ECC algorithm performs the worst, as this algorithm just processed the original data with clustering, and all the protection is provided by the similar attributes of the clustered data set. Therefore, this algorithm cannot get rid of the correlation between the published location and the sensitive location, and the adversary can utilize the correlation attack to guess the highest correlation probability as the particular user and get the privacy.

**Fig 3:**
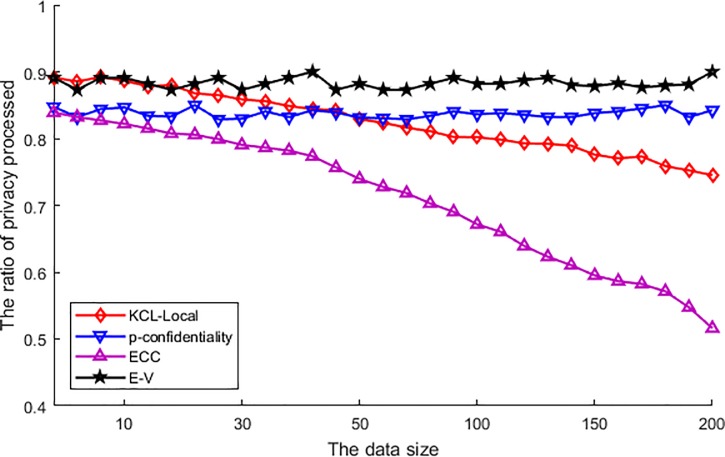
The ratio of privacy processed.

Fig 3 shows the ratio of privacy processed. From this figure, we can see the result that ratio values of different various algorithms are varied. Among these algorithms, the E-V algorithm performs the best. This is because the E-V algorithm has to make the published location has similar correlation probability with sensitive location and transmits all original locations into the centroid location, except that the original location is the centroid. In this procedure, the published data nearly all processed by the algorithm, which made the ratio of privacy processed much higher than the other algorithms, and not affected by the increase of original data. As the conception of p-confidentiality is similar with E-V algorithm, its performance is just a bit worse than E-V. This algorithm utilized an obfuscated region to disturb the correlation of each location, and in the region a large number of original data is mingled to achieve correlation generalization. But as the adjacent locations are hard to distinguish naturally and the obfuscated region failed to process these locations. Thus, the ratio of privacy processed is a bit lower than E-V. Furthermore, this algorithm is also not affected by the increase of original locations similarly. In the other two algorithms, the KCL-Local algorithm is processed the original data with local suppression. The suppression rejected the publishing of the sensitive location, but as the sensitive location is constrained in a finite number, the suppression is unlikely to process a large number of original data. Moreover, as the increase of original data optimized the location generalization, it makes fewer locations need to be suppressed. Therefore, the ratio of privacy processed is lower than above two algorithms, and its ratio of privacy processed is descending with the increase of original data. At last, ECC utilizes the clustering to generalize the location of the user, and utilize the similarity to protect the privacy of the published data. Thus, this algorithm does not process the original data by any special operation and its ratio of privacy processed is the lowest. Furthermore, along with the increasing of original data, similar kinds of location become increasingly, then the clustering become easier to accomplish, which results in the ratio of privacy processed descending with the increase of original data and more serious than KCL-Local algorithm.

**Fig 4:**
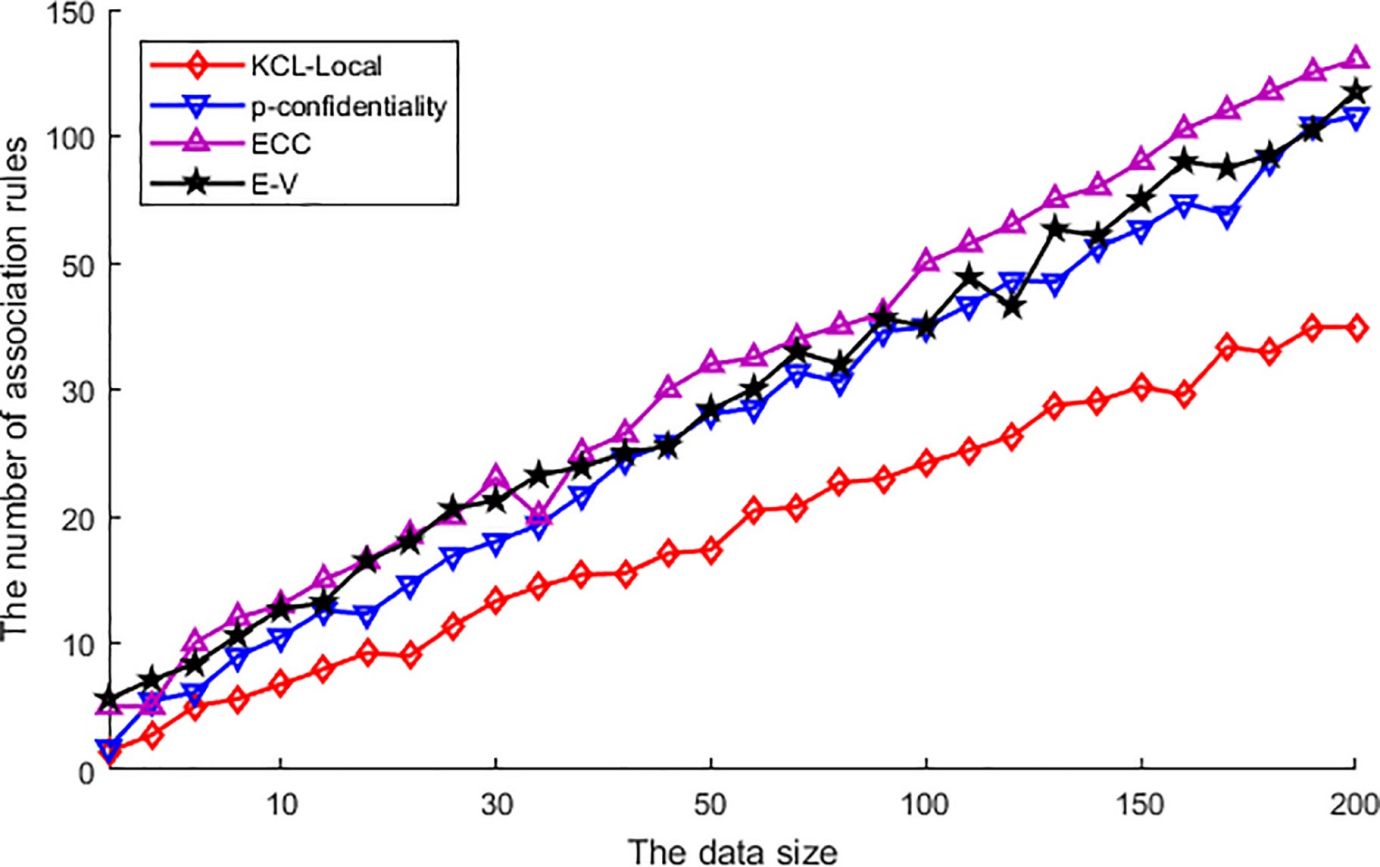
The number of association rules.

In general, the aim of publishing data is to find the available information or association rules, so as to utilize the information or rules to provide better service. Fig 4 shows the number of association rules mining from the published data that processed by the different algorithms. In this figure, the number of association rules is ascending with the increase of published data, as the more published data mean more association rules can be mined. Among these algorithms, the ECC performs best and mines out the maximum number of association rules. This is because the ECC utilizes the clustering to generalize the published data, and protects the privacy with location generalization. Thus, the correlation feature of the original data does not be destroyed during the protection processing, and the association between each attribute can be preserved, so the association rule will be easier to be mined. Other algorithms such as E-V and p-confidentiality, as they utilize a region to process the original data and publish the transmitted or obfuscated data, the process generates the difference between the published data and the original data. Then the correlation between each location will be destroyed in a certain degree, and affects the mining result of association rules. Thus, the performance of E-V algorithm or p-confidentiality algorithm is slightly inferior to ECC. At last, we can see that the performance result of the KCL-Local algorithm is the worst. This is because the KCL-Local has got rid of partially sensitive location in order to protect the publishing data, and the suppression cuts off a large number of correlations between the published locations. Thus, the mining algorithm is difficult to find available information and the number of association rules is affected seriousness.

**Fig 5:**
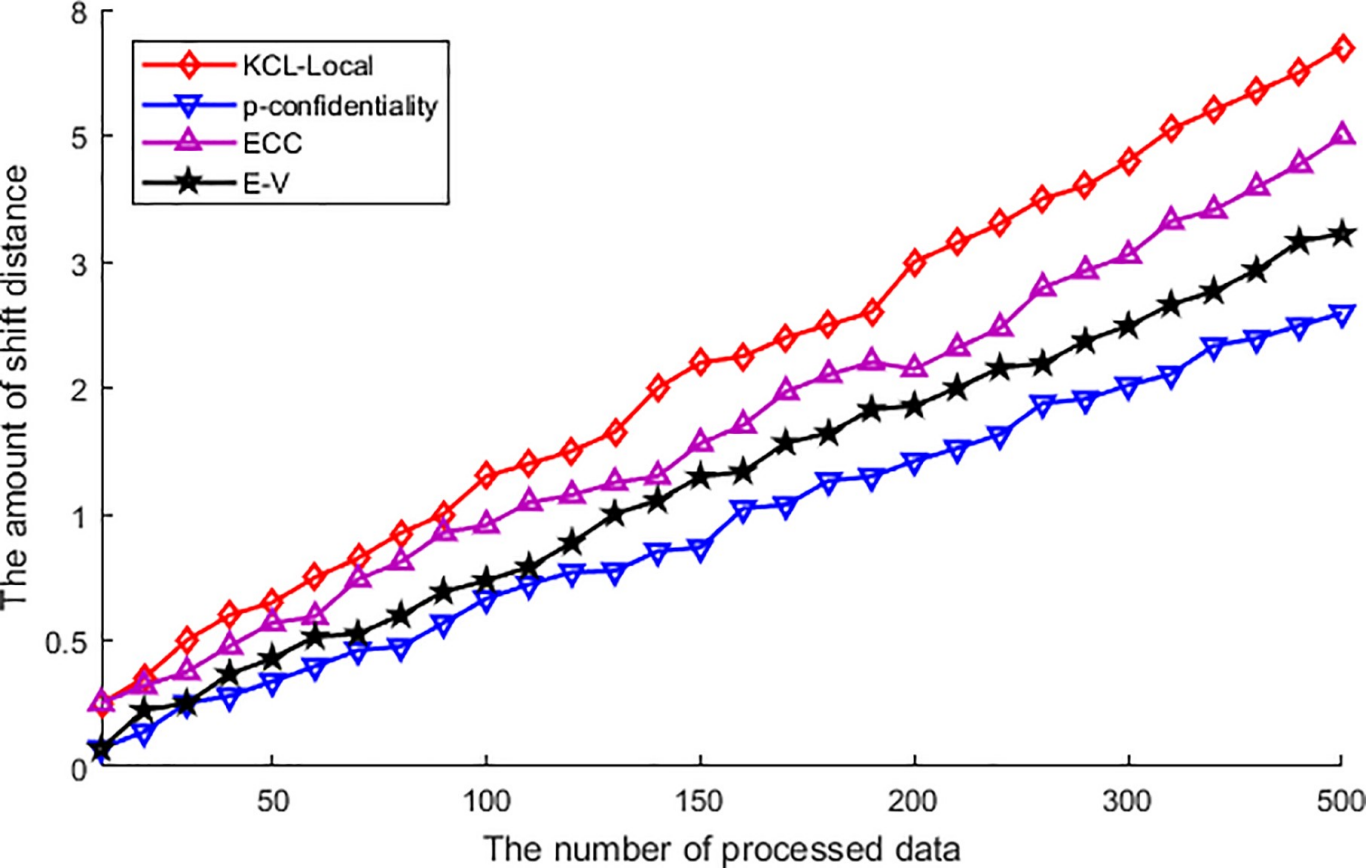
The amount of shift distance.

Fig 5 shows the different values of each algorithm in the evaluation of the amount of shift distance. From this figure, we can see that the shift distance of each algorithm is ascending with the increase of published data. It is because each algorithm has to process the original data to accomplish the privacy protection, and the procedure of privacy protection has to transmit or change the original data to the published data. Thus, the protection procedure has brought about the shift of original data, and the more data been processed the more changing, and then leads to the ascending of shift distance with the increase of published data. Among the performance of various algorithms, the amount of shift distance of KCL-Local algorithm is the maximum. As this algorithm has utilized the suppression and got rid of the sensitive data, and brings about a major difference between the original data and the published data. So based on the calculation method of shift distance, the amount of shift distance is ascending faster than other algorithms along with the increasing of processed data. The ECC algorithm utilizes the clustering to achieve privacy protection. However, the procedure of clustering has a potential defect, and the defect is the discarding of border nodes. Thus, similarly with the KCL-Local algorithm, the discarded border nodes had resulted in the ascending of shift distance along with the increase of processed data, and the amount of shift distance is higher than the other two algorithms. For the other two algorithms, the E-V algorithm performs a bit worse than the p-confidentiality algorithm. This is because the E-V algorithm has transmitted the original data into the coordinate of the vicinity of centroid, and this transmission has changed the position of the original data. But as the feature of voronoi diagram, the shift distance is much minor than data discarding, and then the performance of E-V algorithm is better than the KCL-Local algorithm and the ECC algorithm. At last, the shift distance of p-confidentiality algorithm is the minimum. As this algorithm just utilizes the obfuscated region to disturb the published data, and the obfuscation does not transmit or change any original data. So the amount of shift distance is the minimum, although ascending with the increase of published data.

**Fig 6:**
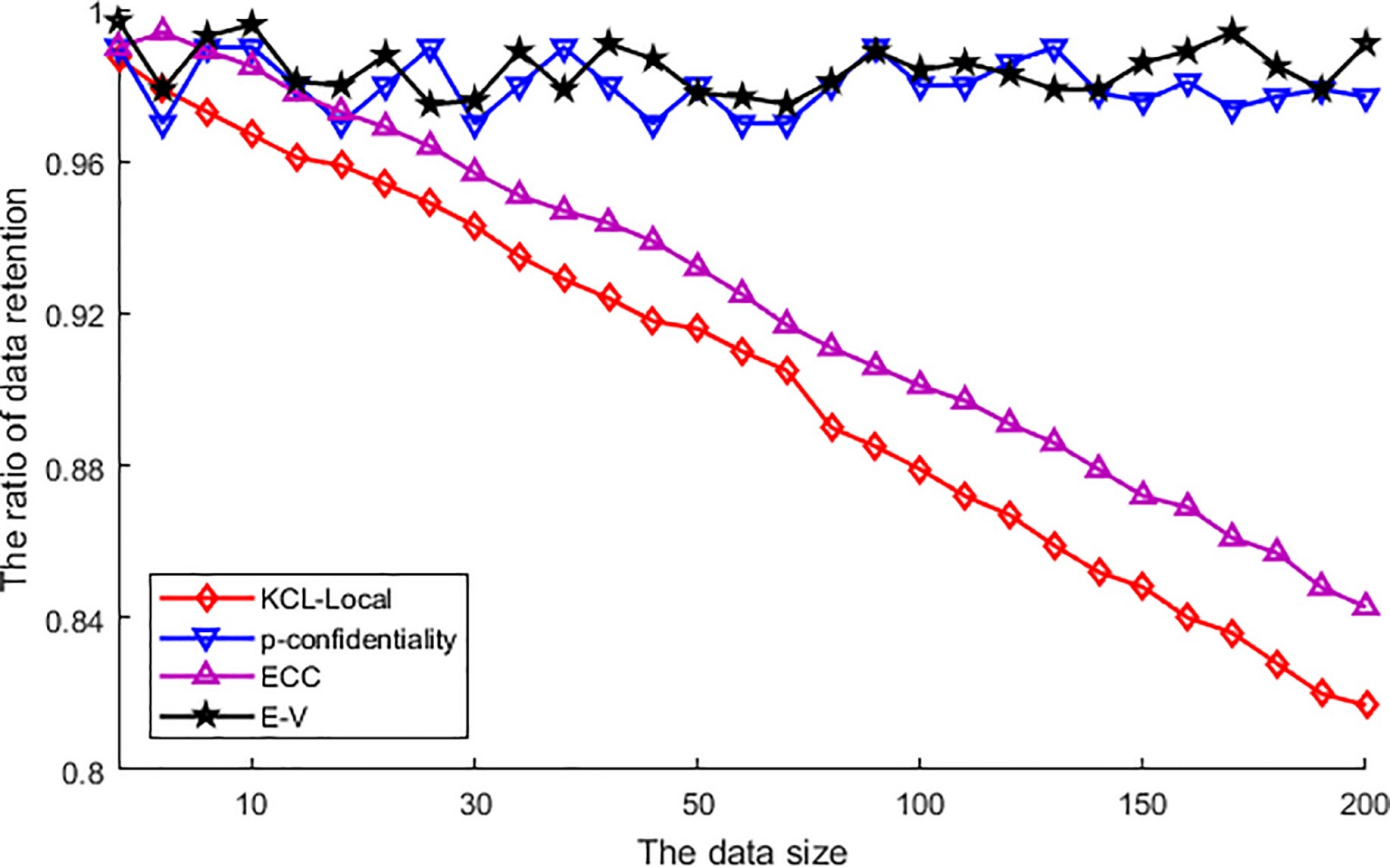
The ratio of data retention.

Fig 6 shows the ratio of data retention. From the comparison of different algorithms, the retention ratio of E-V algorithm is the maximum and does not descend with the increase of data size. As the E-V algorithm just transmits the original data into the coordinate of vicinity centroid, and this algorithm does not get rid of any data during the processing of privacy protection. In addition, the performance of p-confidentiality is similar with E-V algorithm. As the conception of p-confidentiality is utilizing obfuscated data to generalize the published location, and the obfuscation protected the privacy. So this process also does not get rid of any data during the processing of privacy protection in data publishing similarly. In the other two algorithms, the ECC is utilizing clustering to accomplish data generalization. Just as we have inferred in the above section, in order to accelerate the process of data clustering, the clustering has to discard the border nodes. As a result, the more amount of original data the more discarded border nodes, and then the ratio of data retention descending with the increase of the data size. At last, the KCL-Local algorithm utilizes the suppression to process the privacy protection. As a result, the suppression has got rid of a large number of sensitive data. Furthermore, as the more amounts of original data brought about more sensitive data, the suppression has to get rid of more sensitive data, and then the ratio of data retention descending with the increase of the data size. So compared with other three algorithms this algorithm performs the worst.

In conclusion, compared with the other three algorithms in above simulation experiments, we can say that the performance of E-V algorithm is better than the other three algorithms, no matter in the capability of privacy protection or the availability of published data. So our proposed scheme is suitable for application in the realistic environment and has vast potential for future development.

## Conclusion

In recent years, the domain of published data privacy protection has brought about close attention, and a lot of privacy protection scheme has been provided. However, as the diversity of publishing data and the limitation of the proposed schemes, researches on privacy protection still has a long way to go. In this paper, we consider a special type of publishing data called location data, and utilize the feature of voronoi diagram to divide the data region, and then transmit the original data into the coordinate of the centroid vicinity. With this disposition the privacy of any user is transmitted into the non-sensitive correlation data. Furthermore, in order to accomplish the procedure of privacy protection, we utilize the entropy to calculate the similarity of correlation probability between the published location and the sensitive location. Then the similarity is measured by the metric of ε-sensitive correlation indistinguishable, so as to choose the appropriate coordinate in the vicinity of centroid. At last, security analysis is provided to verify the security of E-V algorithm, and accordingly with experimental evaluation to further supports the conclusion of higher privacy protection level as well as better performance on the availability of published data. With the deepening of research and complexity, the future work will be located in the privacy protection publishing of image data and the privacy of real time service in the location based service.

## Supporting information

S1 Table (XLS)

## References

[1] Wang H, Zhang Z, Taleb T. Editorial: Special Issue on Security and Privacy of IoT. World Wide Web. 2018;21(1):1–6. 10.1007/s11280-017-0490-9

[2] Shu J, Jia X, Kan Y, Hua W. Privacy-Preserving Task Recommendation Services for Crowdsourcing. IEEE Transactions on Services Computing. 2018;PP(99):1–.

[3] Sun X, Wang H, Li J, Pei J. Publishing anonymous survey rating data. Data Mining and Knowledge Discovery 2011;3(23):379–406.

[4] Fung BCM, Wang K, Chen R, Yu PS. Privacy-preserving data publishing: A survey of recent developments. ACM Computing Surveys. 2010;42(4):14 10.1145/1749603.1749605 WOS:000279357800002.

[5] Yabo X, Fung BCM, Ke W, Fu AWC, Jian P, editors. Publishing sensitive transactions for itemset utility. Data Mining, 2008 ICDM '08 Eighth IEEE International Conference on; 2008 15–19 Dec. 2008.

[6] Bonchi F, Lakshmanan LVS, Wang H. Trajectory anonymity in publishing personal mobility data. SIGKDD Explor Newsl. 2011;13(1):30–42. 10.1145/2031331.2031336

[7] Sui P, Wo T, Wen Z, Li X, Ieee. Privacy-Preserving trajectory publication against parking point attacks. 2013 IEEE 10th International Conference on and 10th International Conference on Autonomic and Trusted Computing (Uic/Atc) Ubiquitous Intelligence and Computing. 2013:569–74. 10.1109/uic-atc.2013.75 WOS:000346129800078.

[8] Zhao J, Zhang Y, Li XH, Ma JF. A trajectory privacy protection approach via trajectory frequency suppression. Chinese Journal of Computers. 2014;37(10):2096–106.

[9] Wang C, Liu H, Wright K-L, Krishnamachari B, Annavaram M. A privacy mechanism for mobile-based urban traffic monitoring. Pervasive and Mobile Computing. 2015;20(2015):1–12.

[10] Gruteser M, Grunwald D, editors. Anonymous usage of location-based services through spatial and temporal cloaking. Proceedings of the 1st international conference on Mobile systems, applications and services; 2003; San Francisco, California. 1189037: ACM.

[11] Xiao Z, Xu J, Meng X, editors. p-Sensitivity: A semantic privacy-protection model for location-based services. International Conference on Mobile Data Management Workshops; 2008.

[12] Fuyu L, Hua KA, Ying C, editors. Query l-diversity in location-based services. Mobile Data Management: Systems, Services and Middleware, 2009 MDM '09 Tenth International Conference on; 2009 18–20 May 2009.

[13] Wang Y, Xia Y, Hou J, Gao SM, Nie X, Wang Q. A fast privacy-preserving framework for continuous location-based queries in road networks. Journal of Network and Computer Applications. 2015;53(2015):57–73. 10.1016/j.jnca.2015.01.004 WOS:000356184900005.

[14] Lei Z, Chun-guang M, Song-tao Y, Xiao-dong Z. A real-time similar trajectories generation algorithm for trajectories differences identification resistance. Journal of Harbin Engineering University. 2017;2017(07):1173–8.

[15] Ma C, Zhang L, Yang S, Zheng X, Ke P. Achieve personalized anonymity through query blocks exchanging. China Communications. 2016;13(11):106–18.

[16] Chunguang M, Lei Z, Songtao Y, Xiaodong Z. Hiding Yourself Behind Collaborative Users When Using Continuous Location-Based Services. Journal of Circuits, Systems and Computers. 2017;26(07):1750119:1-:25.

[17] Niu B, Zhu XY, Li QH, Chen J, Li H. A novel attack to spatial cloaking schemes in location-based services. Future Generation Computer Systems-the International Journal of Grid Computing and Escience. 2015;49(2015):125–32. 10.1016/j.future.2014.10.026 WOS:000355062700012.

[18] Dargahi T, Ambrosin M, Conti M, Asokan N. ABAKA: A novel attribute-based k-anonymous collaborative solution for LBSs. Computer Communications. 2016;85(2016):1–13. 10.1016/j.comcom.2016.03.002 WOS:000376830900001.

[19] Zhang L, Ma C, Yang S, Zheng X. Probability Indistinguishable: A Query and Location Correlation Attack Resistance Scheme. Wireless Personal Communications. 2017;97(4):6167–87.

[20] Lei Z, Chun-guang M, Song-tao Y, Zeng-peng L. CP-ABE based users collaborative privacy protection scheme for continuous query. Journal on Communications. 2017;38(09):76–85.

[21] Zhang L, Li J, Yang S, Wang B. Privacy Preserving in Cloud Environment for Obstructed Shortest Path Query. Wireless Personal Communications. 2017;96(2):2305–22.

[22] Li Z, Xiang C, Wang C. Oblivious Transfer via Lossy Encryption from Lattice-Based Cryptography. Wireless Communications and Mobile Computing.

[23] Li Z, Ma C, Ding W. Achieving Multi-Hop PRE via Branching Program. IEEE Transactions on Cloud Computing. 2017;PP(99):1-.

[24] Li Z, Ma C, Ding W. Leakage Resilient Leveled FHE on Multiple Bit Message. IEEE Transactions on Big Data. 2017;PP(99):1–.

[25] Chen R, Fung BCM, Mohammed N, Desai BC, Wang K. Privacy-preserving trajectory data publishing by local suppression. Information Sciences. 2013;231(2013):83–97. 10.1016/j.ins.2011.07.035 WOS:000316836600007.

[26] Terrovitis M, Poulis G, Mamoulis N, Skiadopoulos S. Local Suppression and Splitting Techniques for Privacy Preserving Publication of Trajectories. IEEE Transactions on Knowledge & Data Engineering. 2017;29(7):1466–79.

[27] Chow C-Y, Mokbel MF. Trajectory privacy in location-based services and data publication. SIGKDD Explor Newsl. 2011;13(1):19–29. 10.1145/2031331.2031335

[28] Lu Q, Wang C, Xiong Y, Xia H, Huang W, Gong X. Personalized Privacy-Preserving Trajectory Data Publishing. Chinese Journal of Electronics. 2017;26(2):285–91.

[29] Zheng X, Cai Z, Yu J, Wang C, Li Y. Follow But No Track: Privacy Preserved Profile Publishing in Cyber-Physical Social Systems. IEEE Internet of Things Journal. 2017;PP(99):1–.

[30] Lei Z, Lili H, Desheng L, Jing L, Qingfeng J, Qi Y. An Attribute Generalization Mix-Zone Without Privacy Leakage. IEEE Access. 2019;7(1):57088–99. 10.1109/ACCESS.2019.2898996

[31] Li M, Zhu L, Zhang Z, Xu R. Achieving Differential Privacy of Trajectory Data Publishing in Participatory Sensing. Information Sciences. 2017;400–401:1–13.

[32] Cicek AE, Nergiz ME, Saygin Y. Ensuring location diversity in privacy-preserving spatio-temporal data publishing. Vldb Journal. 2014;23(4):609–25. 10.1007/s00778-013-0342-x WOS:000339904500005.

[33] Lin C, Wu GW, Yu CW. Protecting location privacy and query privacy: a combined clustering approach. Concurrency and Computation-Practice & Experience. 2015;27(12):3021–43. 10.1002/cpe.3244 WOS:000358507500009.

[34] Verma P, Boghey R, Rai S, Verma P, Boghey R, Rai S. Classifying Student’s Learning Experience using Improved Apriori and CART. International Journal of Computer Applications. 2017;174(1):34–40.

